# Whey Protein Peptides Self-Assembled Nanoparticles with Intrinsic Cholesterol Esterase Inhibition Enhance Stigmasterol Bioaccessibility and Hypocholesterolemic Effects

**DOI:** 10.3390/nu18172934

**Published:** 2026-09-07

**Authors:** Haoyu Wang, Zhiyuan Ma, Han Gong, Yang Zou, Haijiao Zhang, Xiaohong Chen, Xueying Mao

**Affiliations:** 1 Key Laboratory of Functional Dairy, Ministry of Education, College of Food Science and Nutritional Engineering, China Agricultural University, Beijing 100083, China; s20243061336@cau.edu.cn (H.W.); mazhiyuan@cau.edu.cn (Z.M.); gonghan@cau.edu.cn (H.G.); 2 Key Laboratory of Functional and Flavor Dairy Processing, Ministry of Agriculture, Tianjin Haihe Dairy Co., Ltd., Tianjin 300300, China; 18526628166@163.com (Y.Z.); zhjhhyf123@163.com (H.Z.); xiaohong22392578@163.com (X.C.)

**Keywords:** whey protein peptides, stigmasterol, self-assembled nanoparticles, cholesterol esterase inhibition, bioaccessibility, hypocholesterolemic efficacy

## Abstract

**Background**: Stigmasterol (St) holds promise as a natural cholesterol-lowering agent, yet its poor aqueous solubility and low bioaccessibility severely constrain its application in functional foods. **Methods**: In this study, we exploited whey protein peptides (WPP) with intrinsic cholesterol esterase (CEase) inhibitory activity to construct St-loaded self-assembled nanoparticles. Subsequently, we investigated its physicochemical properties and formation mechanism and evaluated its hypocholesterolemic activity through animal experiments. **Results**: Driven by non-covalent hydrophobic and hydrogen-bonding interactions, the optimized St-loaded WPP nanoparticles (St@WPP) ((234.47 ± 1.36) nm) achieved a high encapsulation efficiency (EE) of 74.73% ± 2.19%. This nano-encapsulation markedly improved St dispersibility and storage stability, and significantly elevated its bioaccessibility from 9.00% ± 0.14% to 19.41% ± 0.69% after simulated gastrointestinal digestion. St@WPP administration effectively ameliorated dyslipidemia, as evidenced by reduced serum total cholesterol (TC) and low-density lipoprotein cholesterol (LDL-C), which may be associated with reduced intestinal cholesterol availability and increased fecal cholesterol content. Moreover, St@WPP alleviated hepatic steatosis, attenuated systemic inflammation, and rebalanced gut microbiota architecture. **Conclusions**: Collectively, the WPP shell functions as a delivery carrier that improves the water solubility and bioaccessibility of St, thereby broadening its potential for application in low-fat or aqueous-based food systems. The resulting St@WPP exhibits notable cholesterol-lowering activity, positioning it as a promising functional food ingredient with potential benefits for cholesterol management.

## 1. Introduction

Abnormal cholesterol metabolism, particularly elevated low-density lipoprotein cholesterol (LDL-C), is a primary driver of hypercholesterolemia and atherosclerotic cardiovascular diseases, which account for approximately 3.9 million deaths annually worldwide [1,2]. Although statins remain the first-line pharmacological intervention by competitively inhibiting the rate-limiting enzyme 3-hydroxy-3-methylglutaryl-coenzyme A reductase (HMGCR) involved in cholesterol synthesis to reduce LDL-C by approximately 30–50%, their long-term use is often associated with adverse effects, including myopathy, hepatorenal toxicity, and new-onset diabetes [3]. This clinical dilemma has intensified the search for natural, safe, and effective bioactive compounds as alternative or adjunctive dietary strategies for cholesterol management.

Disruption of cholesterol homeostasis can result from imbalances in its synthesis, transport, absorption, or excretion [4]. Under normal physiological conditions, excess free cholesterol can be rapidly esterified by acyl-CoA:cholesterol acyltransferase (ACAT) and temporarily stored in intracellular lipid droplets. However, this storage mechanism has limited capacity and is insufficient to cope with chronic cholesterol overload [5]. In an overloaded state, free cholesterol exceeds the esterification capacity of ACAT, leading to abnormal enlargement of lipid droplets and accumulation of unesterified cholesterol on the endoplasmic reticulum membrane. This accumulation disrupts endoplasmic reticulum function and triggers oxidative stress and inflammatory responses [6,7]. Therefore, modulating key genes such as *CYP7A1*, *HMGCR*, and *ABCG5* is essential for inhibiting cholesterol synthesis, decreasing absorption, and promoting fecal excretion, thereby maintaining normal blood cholesterol levels [8].

Dietary cholesterol, contributing approximately 20% to the body’s cholesterol pool, exerts a profound impact on circulating lipid levels [9]. Intestinal cholesterol absorption is critically dependent on the hydrolysis of cholesteryl esters by cholesterol esterase (CEase), a bile salt-activated lipase that liberates free cholesterol for subsequent uptake by enterocytes [10]. Consequently, CEase has emerged as a promising molecular target for curbing hypercholesterolemia at its absorptive source. Notably, milk proteins serve as a valuable reservoir of CEase-inhibitory peptides. For instance, Alcalase-hydrolyzed camel casein peptides have been shown to competitively interfere with CEase–substrate binding, thereby attenuating dietary cholesterol absorption [11]. Additionally, peptides derived from camel whey protein and bovine casein have also exhibited potential in inhibiting CEase activity [12,13].

Parallel to enzymatic inhibition, phytosterols (Ps), including β-sitosterol, campesterol, and stigmasterol (St), offer a second line of defense by competitively displacing cholesterol from intestinal mixed micelles and uptake sites, owing to their structural analogy to cholesterol [14,15]. A meta-analysis has confirmed that a daily intake of 2 g Ps reduces LDL-C by approximately 10% [16]. Among these, St exhibits superior efficacy over β-sitosterol in curtailing cholesterol/bile acid reabsorption and mitigating hepatic lipid deposition [17]. However, the translational potential of St is severely bottlenecked by its high hydrophobicity and exceptionally poor bioaccessibility, which necessitates co-ingestion with high-fat matrices for effective absorption—a significant drawback for application in low-fat or aqueous functional foods [18].

Self-assembled nano-delivery systems based on food-grade biopolymers have been extensively validated to enhance the water dispersibility, stability, and gastrointestinal fate of hydrophobic nutraceuticals [19]. Food-derived proteins and peptides with self-assembling properties are considered ideal materials for constructing such carriers, as their amphiphilic nature facilitates spontaneous self-assembly into micellar architectures via non-covalent forces [20]. Nanoparticles based on sodium caseinate, whey protein concentrate, and zein have been shown to significantly enhance the water solubility and bioaccessibility of Ps [6,21]. Nevertheless, most existing studies treat these peptides merely as inert structural materials. An intriguing yet underexplored paradigm is the dual-functional integration, wherein the peptide-based nanocarrier not only encapsulates the cargo but also contributes its intrinsic bioactivity to the therapeutic outcome.

Herein, we propose a St-loaded nanoparticle system (St@WPP) assembled from whey protein peptides (WPP) with inherent CEase-inhibitory properties. We hypothesized that the WPP carrier would improve the oral delivery performance of St by encapsulating it in an amorphous state via hydrophobic and hydrogen-bonding interactions, thereby enhancing its aqueous solubility and bioaccessibility. Given that WPP also possesses CEase-inhibitory activity, the formulated system may offer advantages beyond conventional solubilization. To test this, we systematically optimized the assembly stoichiometry, characterized the physicochemical properties and self-assembly mechanism, and evaluated the hypocholesterolemic efficacy of St-loaded WPP nanoparticles (St@WPP) in a high-fat-diet-induced hyperlipidemic mouse model, focusing on serum lipid profiles, hepatic steatosis, systemic inflammation, and gut microbiota remodeling. This study provides a proof-of-concept for a functional food-grade nanocarrier and offers a viable formulation strategy to unlock the therapeutic potential of St in managing metabolic disorders.

## 2. Materials and Methods

### 2.1. Materials

Whey protein (purity ≥ 80%) was purchased from Hilmar Cheese Company, Inc. (Hilmar, CA, USA)). Stigmasterol (purity ≥ 90%) was purchased from Macklin Biochemical Technology Co., Ltd. (Shanghai, China). Alcalase^®^ 2.4 L (protease from *B. licheniformis*, ≥2.4 AU/g) was purchased from Novonesis (Tianjin, China). Sodium tetraborate, sodium dodecyl sulfate (SDS), ortho-phtaldialdehyde (OPA), dithiothreitol (DTT), L-serine, bile salt (from pig), and pepsin (≥250 Units/mg solid) were purchased from Solarbio Science & Technology Co., Ltd. (Beijing, China). Pancreatin (trypsin activity ≥ 4000 Units/g solid, amylase activity ≥ 7000 Units/g solid, lipase activity ≥ 4000 Units/g solid), 4-Nitrophenyl butyrate (PNPB), sodium taurocholate and cholesterol esterase (≥60 Units/mg solid) were purchased from Shanghai yuanye Bio-Technology Co., Ltd. (Shanghai, China). Pancreatic lipase (≥125 Units/mg solid, PL) was purchased from Sigma Aldrich (Shanghai, China). Triglyceride (TG), total cholesterol (TC), interleukin-1β (IL-1β), interleukin-6 (IL-6), and tumour necrosis factor-α (TNF-α) assay (ELISA) kits were purchased from Jiangsu Enzyme Exemption Industry Co., Ltd. (Yancheng, China). High-density lipoprotein cholesterol (HDL-C), LDL-C, alanine aminotransferase (ALT) and aspartate aminotransferase (AST) assay (ELISA) kits were purchased from Shenzhen Mindray Bio-Medical Electronics Co., Ltd. (Shenzhen, China). Simvastatin was purchased from Zhejiang Jingxin Pharmaceutical Co., Ltd. (Shaoxing, China). The diets used in the current study were purchased from Research Diets, Inc. (New Brunswick, NJ, USA). All other chemicals utilized were of analytical grade.

### 2.2. Characterization of WPP

#### 2.2.1. Preparation of WPP

CEase-inhibitory peptides were prepared according to the method described by Mudgil et al., with slight modifications [10]. Whey protein was dissolved in deionized water at a concentration of 5% (*w*/*v*) and heated in a water bath at 85 °C for 15 min. After cooling to 55 °C, Alcalase was added at an enzyme/substrate ratio of 3000 U/g. The mixture was incubated at 55 °C under magnetic stirring at 600 rpm for 0.25, 0.50, 0.75, 1, 2, 3, or 4 h, during which the pH was maintained at 8.0 by the addition of 1 M NaOH. To inactivate the enzyme, the resulting solution was heated at 85 °C for 20 min. Afterwards, the solution was adjusted to pH 7.0 and subjected to freeze-drying (LGJ-10, Beijing Tianlin Hengtai Technology Co., Ltd., Beijing, China), resulting in WPP.

#### 2.2.2. Degree of Hydrolysis (DH)

The DH of WPP obtained at different enzymatic hydrolysis times was determined using the method modified from Nielsen et al. [22]. The OPA reagent was prepared by dissolving 7.62 g sodium tetraborate in 150 mL deionized water, adding 200 mg SDS, 4 mL absolute ethanol containing 160 mg OPA, and 88 mg DTT, then adjusting the final volume to 200 mL with deionized water. A mixture of 400 μL WPP (1.5 mg/mL) and 3 mL OPA reagent was allowed to react in the dark for 2 min. Absorbance was then measured at 340 nm using an ultraviolet-visible spectrophotometer (GENESYS 10S, Thermo Fisher Scientific, Shanghai, China). Blank and standard samples were prepared using 400 μL deionized water and L-serine (100 μg/mL), respectively, and processed according to the aforementioned procedure.

DH was calculated according to the following equations:

(1) $$Serine\text{-}{NH}_{2}=\left[\frac{\left({OD}_{sample}-{OD}_{blank}\right)}{\left({OD}_{standard}-{OD}_{blank}\right)}\right]\times 0.9516\;meqv/L\times \left[\frac{0.1\times 100}{X\times P}\right]$$

(2) $$h=\frac{\left(Serine\text{-}{NH}_{2}-\beta \right)}{\alpha }$$

(3) $$DH\;\left(\%\right)=\frac{h}{h_{tot}}\times 100$$

where *X* is the weight of the sample, *P* is the percentage of protein in the sample and 0.1 represents the sample volume. α is 1.00 and β is 0.40. *h_tot_* is the protein hydrolysis constant, which has a value of 8.8 for whey protein.

#### 2.2.3. Inhibition of CEase

The CEase inhibitory activity of WPP, obtained at different enzymatic hydrolysis times, was evaluated as previously described [11]. Briefly, 25 μL of each sample (4 mg/mL) was sequentially mixed with 25 μL of *p*-nitrophenyl butyrate (5 mM, dissolved in acetonitrile), 25 μL of sodium phosphate buffer (0.1 M, pH 7.04) containing 5.16 mM sodium taurocholate, and 100 μL of NaCl buffer (100 mM). The reaction was initiated by the final addition of 25 μL of porcine pancreatic CEase (5 μg/mL). After incubation at 37 °C for 30 min, the absorbance was measured at 405 nm using a microplate reader (iMark, Bio-Rad Laboratories (Shanghai) Co., Ltd., Shanghai, China). The percentage of enzyme inhibition was calculated according to the following equation:

(4) $$CEase\;Inhibition(\%)=\left[1-\frac{\left(C-D\right)}{\left(A-B\right)}\right]\times 100$$

where *A* is the absorbance of the control, *B* is the absorbance of the control blank, *C* is the absorbance of the sample, and *D* is the absorbance of the sample blank. These values correspond to the absorbance values of wells containing enzyme and buffer, buffer alone, enzyme and sample, and buffer and sample, respectively. Substrate was present in all reactions.

#### 2.2.4. Molecular Docking of WPP with CEase

The sequences of WPP were analyzed using liquid chromatography–tandem mass spectrometry. The potential toxicity, allergenicity, and human intestinal absorption of the identified peptides were evaluated in silico (for details, see Supplementary Materials).

The 3D structure of CEase (PDB ID: 1CLE) was obtained from the RCSB PDB database. The protein receptor results were then imported into Discovery Studio 2019 Client software. Water molecules and endogenous ligands were removed, incomplete amino acid residues were supplemented, and hydrogen atoms were added before molecular docking. The 3D structures of the identified peptides were constructed in Discovery Studio, followed by geometry optimization and energy minimization using CHARMM as the force field. A semi-flexible docking approach (CDOCKER) was used to investigate the molecular interactions between the peptides and CEase, with the binding site defined by endogenous cholesteryl linoleate ligands (coordinates: *x* = 2.773, *y* = −14.194, *z* = −5.228, radius = 12.000). Default values were used for the remaining molecular docking settings. Docking results were ranked according to the -CDOCKER Energy score, with higher scores indicating a stronger binding strength.

#### 2.2.5. Critical Micelle Concentration (CMC)

The CMC was determined using the method described by James and Mandal [23]. The 2 h WPP was dissolved in deionized water at concentrations ranging from 0.02 to 0.32 mg/mL, and the conductivity of these solutions was determined at 25 °C. The CMC was determined by fitting two linear regression lines to the conductivity-concentration data, corresponding to the premicellar and postmicellar regions, respectively. The CMC value was taken as the intersection point of these two fitted lines.

### 2.3. Preparation of St@WPP

The method was modified based on the approach reported by Wang et al. [24]. Briefly, St (8 mg/mL, dissolved in ethanol) was added dropwise at a rate of 5 mL/min to 2 h WPP (20 mg/mL) under stirring at St-to-WPP volume ratios of 1:5, 2:5, 4:5, and 1:1. A 100 mL aliquot of each resulting mixture was then sheared at 10,000 rpm for 10 min using a shear mixer (FM200, FLUKO, Shanghai, China), followed by ultrasonication at a frequency of 25 kHz and an output power of 250 W using a cell disruptor (SCIENTZ-IID, Ningbo Scientz Biotechnology Co., Ltd., Ningbo, China) with a pulse mode of 3 s on and 3 s off for a total processing time of 90 s. Both shearing and ultrasonication were conducted in an ice bath to maintain the sample temperature. Ethanol was subsequently removed completely using a rotary evaporator (RE-2000B, Shanghai, China) at 45 °C. After freeze-drying, St@WPP powders with mass ratios of 1.6:20, 3.2:20, 6.4:20, and 8.0:20 (St to WPP) were obtained.

### 2.4. Characterization of St@WPP

#### 2.4.1. Particle Size and Zeta Potential

The particle size and zeta potential of St@WPP at different mass ratios were measured using a dynamic light scattering (DLS) instrument (Zetasizer Nano ZS-90, Malvern, UK) with a scattering angle of 90° at 25 °C. Parameters: solvent viscosity = 0.887 mPa·s, refractive index of the medium = 1.330, material refractive index = 1.450. Prior to measurement, the stock dispersion (20 mg/mL) was diluted 50-fold with deionized water to achieve an attenuation of 7. The Z-average diameter was recorded as the mean particle size. All measurements were performed on three independent batches.

#### 2.4.2. Encapsulation Efficiency (EE) and Loading Capacity (LC)

The EE and LC of St@WPP at different mass ratios were determined using a sulfate-phosphate-ferric method, as described by Feng et al. [6]. St@WPP powder (100 mg) was dissolved in 5 mL of deionized water and centrifuged (SF-TGL-18R, Shanghai, China) at 500× *g* for 5 min at 4 °C, discarding the supernatant. Ethyl acetate was added to dissolve any unencapsulated St in the precipitate, followed by another centrifugation. The resulting supernatant was collected for analysis. The working solution was prepared by diluting 1.5 mL 10% (*w*/*v*) FeCl_3_ solution (in 85% phosphoric acid) to a final volume of 100 mL with concentrated sulfuric acid. After mixing 2 mL sulfuric-phosphoric-ferric reagent was mixed with 4 mL sample, absorbance was measured at 520 nm after a 15 min reaction, and the concentration was calculated according to a standard curve.

The EE and LC were calculated using the following equations:

(5) $$EE\;(\%)=\frac{\left(S_{0}-S_{1}\right)}{S_{0}}\times 100$$

(6) $$LC(g/100\;g)=\frac{\left(S_{0}-S_{1}\right)}{M}\times 100$$

where *S*_0_ is the initial weight of St before loading, *S*_1_ is the weight of free St after loading into solution, and M is the total weight of the nanoassemblies.

#### 2.4.3. Storage Stability

For stability assessment, St, WPP, and St@WPP powder were dissolved in deionized water and stored at 4 °C for 21 days. The visual appearance of the samples was recorded on days 1, 5, 9, 13, 17, and 21 to assess water dispersibility and stability during storage.

#### 2.4.4. In Vitro Simulated Digestion and Bioaccessibility of Stigmasterol

Free St and St@WPP samples (containing St at an initial concentration of 6.4 mg/mL) underwent two-stage in vitro digestion according to the INFOGEST protocol [25] (for details, see Supplementary Materials). Briefly, for the gastric phase, the sample was mixed with the enzyme-containing simulated gastric fluid at a 1:1 volume ratio, yielding final concentrations of 2000 U/mL pepsin and 0.075 mM Ca^2+^. The mixture was incubated at 37 °C for 2 h with the pH maintained at 3.0. For the intestinal phase, the gastric digesta was mixed with the enzyme-containing simulated intestinal fluid at a 1:1 volume ratio. The amount of pancreatin added was standardized to achieve a final trypsin activity of 100 U/mL in the digestion mixture. For lipid digestion, additional pancreatic lipase was supplemented to reach a final lipase activity of 2000 U/mL. The final mixture also contained 10 mM bile salts and 0.3 mM Ca^2+^. The intestinal mixture was further incubated at 37 °C for 2 h with the pH maintained at 7.0. After digestion, the samples were centrifuged at 2500× *g* for 20 min at 4 °C. The supernatants were considered as the aqueous bioaccessible fraction [26]. The St content in the supernatant was assessed using the sulfate-phosphate-ferric method, as described in Section 2.4.2. Bioaccessibility of St was subsequently calculated according to the following equation:

(7) $$Bioaccessibility(\%)=\frac{C_{1}}{C_{0}}\times 100$$

where *C*_0_ is the concentration of St in the original digestive phase and *C*_1_ is the concentration of St in the micelle fraction after two-stage digestion.

### 2.5. Self-Assembly Mechanism and Morphology of St@WPP

#### 2.5.1. Fourier-Transform Infrared (FTIR) Spectral Analysis

FTIR (Nicolet iS 10, Thermo Fisher Scientific, Waltham, MA, USA) was used to analyze the intermolecular interactions in St@WPP. Dried samples of St, WPP and St@WPP were mixed with KBr at a ratio of 1:100 and pressed into thin slices. The spectra were obtained in the range of 500–4000 cm^−1^ with a resolution of 4 cm^−1^ in transmittance mode.

#### 2.5.2. X-Ray Diffraction (XRD) Analysis

The XRD pattern of St@WPP was obtained at room temperature using a diffractometer (D8 Advance, Bruker, Billerica, MA, USA), scanning from 5° to 40° at a rate of 5°/min in the 2θ range. The operating voltage and filament current were set at 40 kV and 40 mA, respectively. St and WPP were used as controls.

#### 2.5.3. Morphology Analysis

The freeze-dried St@WPP samples were fixed on conductive tape and coated with 30 nm of gold by sputtering for 45 s. The imaging parameters for the scanning electron microscope (SEM, Sigma 300, Carl Zeiss AG, Oberkochen, Germany) were set to an accelerating voltage of 5.0 kV and a magnification of 40,000×.

The sample solution (10 μL) was dropped onto a 200-mesh carbon–copper grid and stained with 2% (*v*/*v*) phosphotungstic acid. The morphology of St@WPP was monitored using transmission electron microscopy (TEM, TECNAI G2 SPIRIT BIO, Fei, Hillsboro, OR, USA) under an acceleration voltage of 200 kV after the samples were air-dried.

### 2.6. Animal Experimental Design

Forty healthy male C57BL/6J mice (21.94 ± 0.19 g, five weeks old) were purchased from Beijing Vital River Laboratory Animal Technology Co., Ltd. (Certificate No. SCXK-jing-2023-0049, Beijing, China). The mice were housed under standard conditions (20–22 °C, 40–60% humidity, 12/12 h light-dark cycle) and allowed to access food and water ad libitum. All animal experiments were in accordance with the National Research Council’s Guide for the Care and Use of Laboratory Animals and were approved by the Animal Ethics Committee of China Agriculture University (approval number Aw11704202-4-1).

After a seven-day adaptation period on a normal diet (10 kcal% fat, D12450J, Table S2), thirty mice were randomly selected and fed a high-fat diet (HFD, 60 kcal% fat, D12492, Table S2) for 8 weeks. The remaining ten mice served as the normal control (NC) group (*n* = 10). The sample size of 10 mice per group was determined based on previous studies, which demonstrated that this sample size was sufficient to detect statistically significant differences [27,28]. Mice were randomly assigned to groups using a random number table method and housed at five mice per cage, with two cages randomly allocated to each group. Subsequently, the thirty high-fat diet mice were randomly divided into three groups (*n* = 10): the high-fat diet group (HFD), the St-loaded WPP nanoparticles group (St@WPP, 528 mg/kg) and the positive control group (Simvastatin, 5 mg/kg). The NC group was maintained on a normal diet, while other groups were fed with high-fat diet for 9 weeks. During this period, the St@WPP (0.1 mL/10 g, prepared in physiological saline) and Simvastatin groups were given the corresponding dose of St@WPP or simvastatin by gavage (0.1 mL/10 g b.w.), while the NC and HFD groups were given equal volume of saline daily. To minimize spatial and temporal biases, cage positions were rotated daily, and sample collections were performed in a balanced order. The doses of 528 mg/kg/day for St@WPP (St:WPP = 6.4:20, *w*/*w*, containing St 128 mg/kg/day) corresponds to the recommended daily intake of 0.8 g of stigmasterol for adults (60 kg), with the WPP component aligning with the commonly used gavage dose of 400 mg/kg. A meta-analysis indicated that a daily intake of 0.5–1 g of Ps can reduce LDL-C by more than 0.2 mmol/L [29].

At the end of the trial, after overnight fasting, all mice were anesthetized with 2% isoflurane to minimize suffering and then euthanized via cervical dislocation. Blood, feces, liver, and adipose tissue samples were collected for further analysis.

Food intake and body weight were recorded weekly. Three mice were randomly selected from each of the two cages per group, providing 6 mice per group for subsequent biochemical, histopathological, and gut microbiota analyses. All sample analyses were conducted in a blinded manner using coded samples.

### 2.7. Effects of St@WPP on High-Fat-Diet-Fed Mice

#### 2.7.1. Body Weight and White Adipose Tissue Mass

All mice were weighed weekly, and weight gain during the gavage intervention period was calculated. After euthanizing the mice, the weights of inguinal white adipose tissue (iWAT), epididymal white adipose tissue (eWAT) and perirenal white adipose tissue (pWAT) were measured.

#### 2.7.2. Determination of Fecal Cholesterol Levels

The feces were homogenized with absolute ethanol (1:9, *w*/*v*) in an ice-water bath and then centrifuged at 2500 rpm for 10 min. The supernatant was collected. Afterward, fecal cholesterol content was measured using the TC assay kit (Nanjing Jiancheng Bioengineering Research Institute, Nanjing, China).

#### 2.7.3. Biochemical Analysis of Serum and Liver

Serum was obtained by centrifuging blood samples at 3000 rpm for 10 min at 4 °C. The serum levels of TC, TG, HDL-C, LDL-C, ALT, AST, TNF-α, IL-6, and IL-1β were measured using ELISA kits.

Liver tissue was homogenized with a homogenization medium (1:9, *w*/*v*) under ice-water bath conditions. The mixture was then centrifuged at 10,000 rpm for 10 min at 4 °C. The concentrations of TG and TC in the liver homogenate were measured using ELISA kits.

#### 2.7.4. Histopathological Analysis

The liver and epididymal white adipose tissue were fixed in 4% (*v*/*v*) paraformaldehyde for 24 h, followed by preparation of paraffin-embedded sections or frozen sections. These sections were stained with hematoxylin or eosin (H&E) and Oil Red O for histopathological examination. One section and four random fields were examined per mouse by a blinded observer, with field scores averaged per animal (*n* = 6 per group) for statistical analysis. The degree of hepatic steatosis was graded from 0 to 4 based on the average proportion of fat-accumulating hepatocytes observed (Grading 0 is 0–5%, 1 is 6–25%, 2 is 26–50%, 3 is 51–75%, and 4 is 76–100%) [30]. ImageJ 1.54g software was used to measure the average size of fat vacuoles in the liver and the diameter of adipocytes in eWAT.

#### 2.7.5. Gut Microbiota Analysis

Fecal samples were extracted using a fecal DNA kit (Omega Bio-Tek, Norcross, GA, USA), and the extracted DNA was quantified with a micro spectrophotometer (NanoDrop2000, Thermo Fisher Scientific, Waltham, MA, USA). Universal primers for the 16S rDNA conserved region were designed: 5′-ACTCCTACGGGAGGCAGCAG-3′ (universal primer 338F) and 5′-GGACTACHVGGGTWTCTAAT-3′ (universal primer 806R) to amplify the variable region (V3 + V4) or specific gene fragments. Purified amplicons were pooled in equimolar amounts and paired-end sequenced on an Illumina Nextseq 2000 platform (Illumina, San Diego, CA, USA) according to standard protocols by Majorbio Bio-Pharm Technology Co., Ltd. (Shanghai, China). Raw sequencing data were processed using the QIIME2 pipeline (v2022.2). After quality filtering, denoising, and chimera removal via the DADA2 plugin, high-quality amplicon sequence variants (ASVs) were generated. Taxonomic assignment was performed against the SILVA database (v 138) at 99% similarity. To ensure comparability across samples, all samples were rarefied to 20,000 reads per sample, with an average Good’s coverage of 99.09% after rarefaction. Species composition of the samples was analyzed through species annotation and abundance analysis. Alpha diversity, beta diversity and significance were used to assess differences between the samples.

#### 2.7.6. Short-Chain Fatty Acids Content Analysis

The fecal sample was homogenized with 0.5% phosphoric acid at a 20:1 (*w*/*v*) ratio, vortexed for 10 min, and ultrasonicated for 5 min. After centrifugation at 12,000 rpm for 10 min at 4 °C, 100 μL of the supernatant was mixed with 500 μL of MTBE containing 4-methylvaleric acid as the internal standard, vortexed for 3 min, and ultrasonicated for 5 min. This mixture was centrifuged again at 12,000 rpm for 10 min at 4 °C. The supernatant was then injected into a triple quadrupole gas chromatography-mass spectrometer (8890-7000D, Agilent, Santa Clara, CA, USA) equipped with an Agilent VF-WAXms capillary column (25 m × 250 μm × 0.2 μm). Helium was used as the carrier gas at a flow rate of 2 mL/min, with an inlet temperature of 180 °C and a septum purge flow of 3 mL/min. The column temperature program was as follows: initial temperature 40 °C, held for 2 min; ramped to 100 °C at 5 °C/min; then ramped to 230 °C at 15 °C/min and held for 5 min; followed by a post-run at 230 °C for 2 min. The ion sources were set to 230 °C, and full-scan mode was used over a mass range of *m*/*z* 30–1000 at a scan rate of 3.2 scans/s. Short-chain fatty acid (SCFA) concentrations were determined by interpolation from standard curves using the GC-MS Agilent analysis software (version B.09.00).

### 2.8. Statistical Analysis

The results of animal experiments were expressed as mean ± SEM, while other results were presented as mean ± SD. Normality of data distribution was verified using the Shapiro–Wilk test, and homogeneity of variance was confirmed by Levene’s test. For data that met the assumptions of normality and homogeneity of variance, one-way analysis of variance was performed, followed by Duncan’s multiple range test. For non-normally distributed and ordinal data (e.g., steatosis scores), statistical differences were analysed using the Kruskal–Wallis test, followed by Dunn’s post hoc test, and were presented as median (IQR). Body weight data, measured weekly (weeks 0–9 for modeling, weeks 10–18 for treatment), were analyzed using two-way repeated-measures ANOVA with group and time as factors. Mauchly’s test was used to check sphericity, and the Greenhouse–Geisser correction was applied when necessary. Pairwise comparisons were adjusted using Bonferroni’s method. The difference was considered significant at *p* < 0.05. All statistical computations and analyses were performed using SPSS software (Version 26.0). Principal coordinate analysis (PCoA) was performed using the vegan v2.5-3 package, and PERMANOVA (999 permutations, based on Bray–Curtis dissimilarity) along with PERMDISP were used to test overall compositional differences and assess homogeneity of variances among groups. For differential abundance analysis of microbial taxa across multiple groups, the Kruskal–Wallis test was used, followed by Dunn’s post hoc test with FDR correction.

## 3. Results and Discussion

### 3.1. Preparation of Whey Protein-Derived CEase Inhibitory Peptides

#### 3.1.1. Hydrolysis Degree and CEase Inhibitory Profiling of WPP

**Figure 1:**
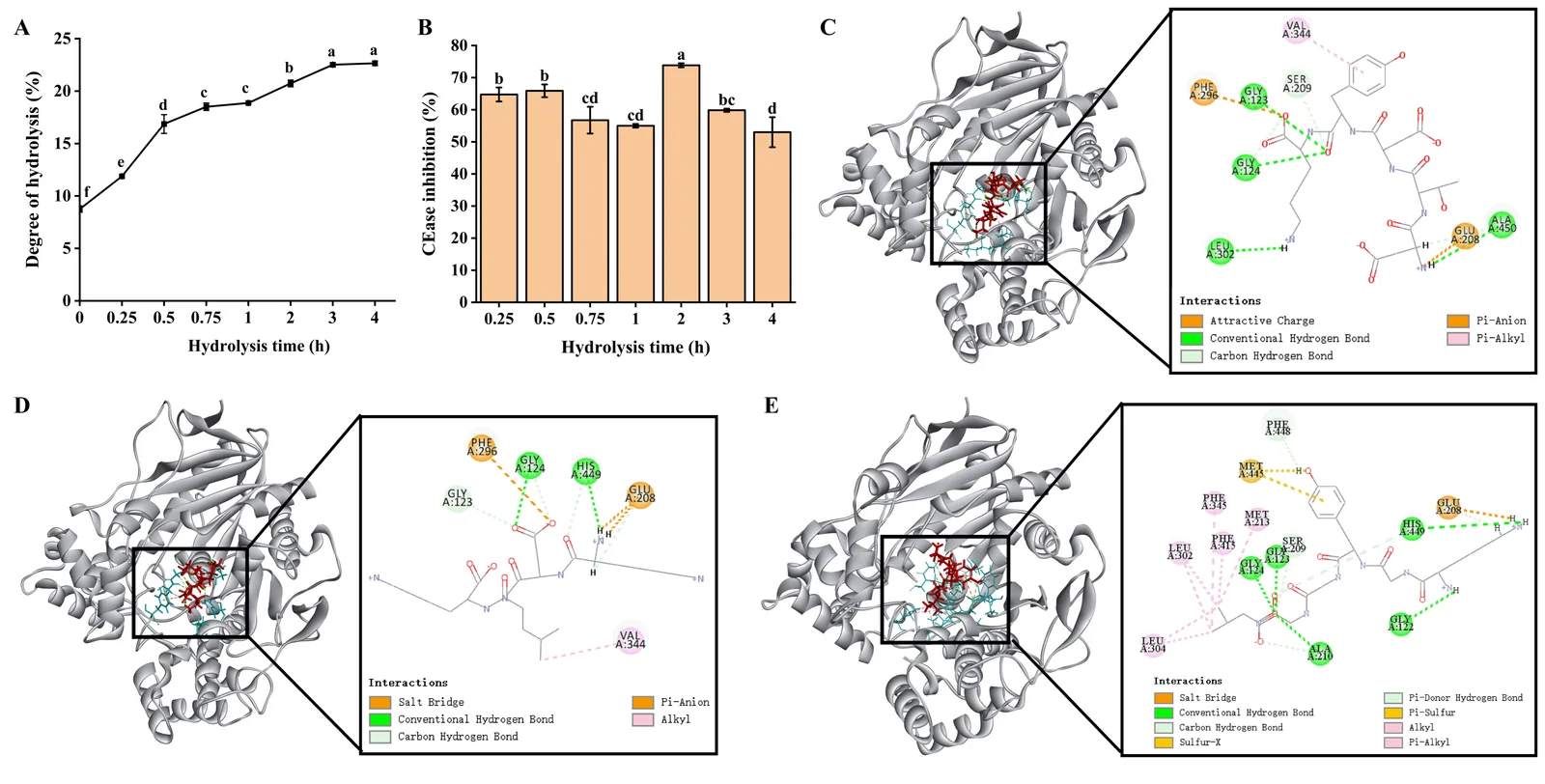
Preparation and characterization of WPP with CEase inhibitory activity. Effects of hydrolysis time on hydrolysis degree (**A**) and CEase inhibition rate (**B**) of WPP. Docked poses and interactions of CEase with (**C**) DTDYK, (**D**) KDLK and (**E**) KGYGGV. Data are presented as mean ± SD, *n* = 3. Different lowercase letters indicate significant differences (*p* < 0.05).

The degree of hydrolysis is a critical indicator for protein hydrolysis extent, influencing peptide chain length and subsequent functional properties. As depicted in Figure 1A, DH exhibited a rapid initial increase within the first 45 min of Alcalase treatment, followed by a gradual plateau, ultimately reaching 22.66% ± 0.22% at 4 h—a kinetic profile consistent with earlier findings [31]. The absence of a significant difference between 3 h and 4 h suggests that hydrolysis approached a plateau.

Notably, CEase-inhibitory activity did not correlate monotonically with DH (Figure 1B). The 2 h hydrolysate exhibited the most potent inhibition (73.85% ± 0.64%), whereas prolonged hydrolysis to 4 h significantly reduced the activity to 53.00% ± 4.67% (*p* < 0.05). This non-monotonic response suggests a temporal sequence of peptide release: Alcalase initially liberates surface-exposed bioactive sequences, which are subsequently degraded upon extended cleavage of internal peptide bonds. Notably, this optimal activity substantially surpassed that reported for adzuki bean peptides (17.15–36.12%) and camel whey pepsin hydrolysates (21.3% ± 0.2%) [32,33]. These results suggest that whey protein is a promising precursor for CEase-targeting bioactive peptides, and thus the 2 h WPP was selected for subsequent nanoparticle fabrication, leveraging its intrinsic CEase-inhibitory capacity as an active carrier. However, it should be noted that these results were obtained using a general esterase substrate (PNPB), which may not fully represent the specific hydrolysis of cholesteryl esters.

#### 3.1.2. Identification and Molecular Docking of Potential CEase Inhibitory Peptides

To further elucidate the interaction mechanism between WPP and CEase, peptide sequence identification, property prediction, and molecular docking were conducted. The identified peptides were predicted to be non-toxic, non-allergenic, and well-absorbed in the intestine (Table S3). Among the identified peptides, DTDYK, KDLK, TMKGL, KGYGGV, and KIDAL exhibited high binding scores (-CDOCKER ENERGY > 80 kcal/mol) and displayed typical characteristics of cholesterol-lowering peptides, namely the presence of acidic amino acids (glutamic acid, aspartic acid and their amides) or hydrophobic amino acids (glycine, valine, leucine, and alanine), suggesting strong cholesterol-lowering potential [34,35]. As shown in Figure 1C–E and Table S3, these five potential CEase inhibitory peptides interacted with residues at the active sites of CEase, including the catalytic triad (Ser209-His449-Glu341) and the oxyanion hole (Gly123, Gly124, and Ala210), as well as nearby residues (e.g., Gly122, Glu208, Leu302, Phe345, Phe415) through hydrogen bonds, hydrophobic interactions, and salt bridges [36]. These interactions may alter the enzyme’s conformation, reduce substrate affinity, and prevent substrate access to the active site, thereby inhibiting CEase activity [37]. It should be noted that the molecular docking analysis is a computational prediction only, and the identified peptides should be considered as potential CEase inhibitors.

### 3.2. Preparation and Characterization of St@WPP

#### 3.2.1. CMC of WPP

**Figure 2:**
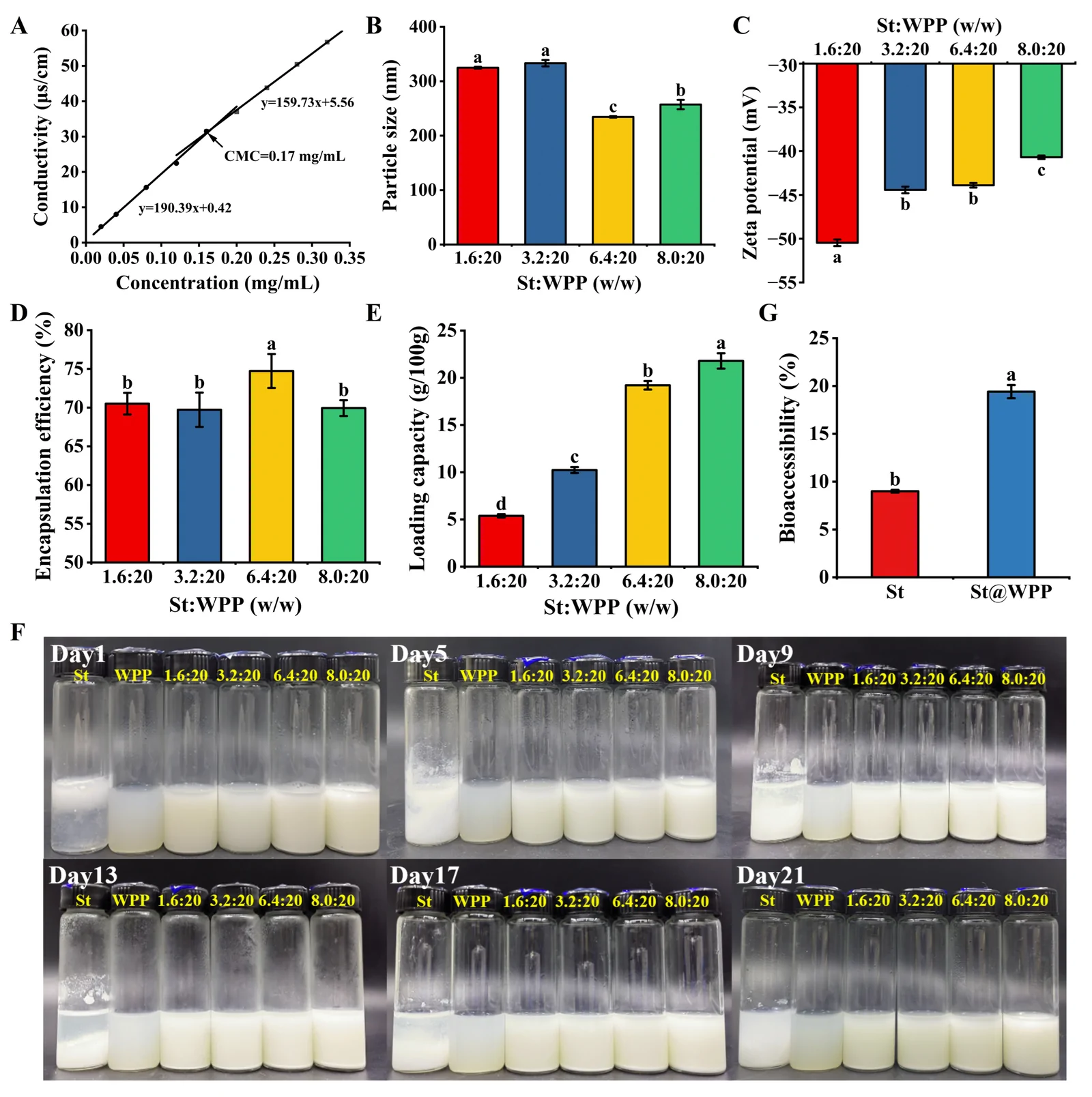
Preparation and characterization of St@WPP. (**A**) The CMC of WPP. (**B**) Particle size, (**C**) zeta potential, (**D**) encapsulation efficiency, (**E**) loading capacity, and (**F**) storage stability of St@WPP at different St:WPP mass ratios. (**G**) The bioaccessibility of St after in vitro digestion. Data are presented as mean ± SD (*n* = 3). Different lowercase letters indicate significant differences (*p* < 0.05).

Amphiphilic WPP, comprising both hydrophobic and hydrophilic domains, spontaneously self-assemble into stable micellar architectures in aqueous media, a process primarily governed by hydrophobic interactions, electrostatic forces, and hydrogen bonding [38]. The CMC of WPP, determined via conductivity measurements (Figure 2A), was established at 0.17 ± 0.00 mg/mL—the inflection point where conductivity increment slows due to micellization-induced reduced ionic mobility. This value is considerably lower than those reported for pea protein (0.73 mg/mL), corn zein (0.81 mg/mL), and wheat glutenin (0.35 mg/mL) peptides [39,40,41]. Such a relatively low CMC of WPP reflects a strong thermodynamic propensity for micellization, which may be advantageous for maintaining colloidal integrity under gastrointestinal dilution, where low CMC carriers resist premature disassembly.

#### 3.2.2. Particle Size and Zeta Potential of St@WPP

Particle size and surface charge are critical determinants of nanocarrier stability and in vivo performance. As depicted in Figure 2B,C, the hydrodynamic diameter of St@WPP varied considerably with the St-to-WPP mass ratio, ranging from a minimum of 234.47 ± 1.36 nm (at a mass ratio of 6.4:20) to a maximum of 333.17 ± 5.85 nm (at 3.2:20), consistent with the compositional dependence reported by Zhao et al. [42]. The corresponding polydispersity index (PDI) values ranged from 0.12 to 0.28 (Table S4), suggesting a relatively narrow size distribution across all formulations. At St-to-WPP mass ratios of 1.6:20, 3.2:20, 6.4:20, and 8.0:20, the zeta potentials of St@WPP were −50.47 ± 0.38 mV, −44.43 ± 0.38 mV, −43.90 ± 0.26 mV, and −40.70 ± 0.20 mV, respectively, indicating that all formulations exhibited highly negative zeta potentials (−50.47 to −40.70 mV), with absolute values well exceeding the conventional stability threshold of ±30 mV, ensuring robust electrostatic repulsion against particle aggregation [43]. Notably, increasing St loading progressively reduced the absolute zeta potential. This trend plausibly arises from hydrogen bonding between the hydroxyl groups of St and carboxyl moieties of WPP, which promotes inward sequestration of negative charges toward the micellar core, thereby diminishing surface charge density [44].

#### 3.2.3. EE and LC of St@WPP

The EE and LC of St@WPP at various mass ratios are summarized in Figure 2D,E. All formulations achieved EE values exceeding 65%, with LC progressively increasing as the St proportion rose—indicating that ultrasonication-assisted assembly, coupled with an appropriate core-to-wall stoichiometry, efficiently entraps St within the WPP matrix. Strikingly, at a St-to-WPP ratio of 6.4:20, the system attained a maximum EE of 74.73% ± 2.19%, with an LC of 19.21 ± 0.45 g/100 g, which was higher than those previously reported phytosterol-loaded carriers, such as whey protein isolate/gum arabic coacervate microcapsules (EE ~59.26%) and whey protein isolate microcapsules (LC ~8.15 g/100 g) [45,46]. Based on these results, the St@WPP system at 6.4:20 was selected as the optimal formulation for subsequent investigations.

#### 3.2.4. Storage Stability of St@WPP

Upon rehydration, St@WPP formed a homogeneous and opalescent dispersion, whereas free St exhibited pronounced aggregation and poor water dispersibility (Figure 2F), suggesting that micellization improves St solubility. The physical stability of St@WPP nanoparticles was preliminarily assessed by visual observation. During 21-day storage at 4 °C, free St gradually sedimented at the vial bottom, while all St@WPP formulations remained macroscopically stable without visible phase separation. This enhanced stability can be attributed to the nanoscale dimensions, which minimize gravitational settling, and the strong electrostatic repulsion conferred by the negative surface charge, which maintains interparticle spacing and prevents aggregation [45,47]. The negligible sediment observed in St@WPP samples likely originates from a minor fraction of unencapsulated St. Collectively, these data demonstrate that St@WPP nanoparticles retain excellent physical stability for at least 21 days after preparation.

#### 3.2.5. Bioaccessibility of Stigmasterol

The intestine is the primary site where St exerts its cholesterol-lowering effects. Bioaccessibility, defined as the proportion of St solubilized in the mixed micellar phase following simulated gastrointestinal digestion, serves as a predictive indicator of its absorbable fraction. As shown in Figure 2G, free St exhibited a remarkably low bioaccessibility of merely 9.00% ± 0.14% after 4 h of sequential digestion, whereas encapsulation within WPP nanoparticles significantly increased this value to 19.41% ± 0.69%, a more than 2-fold enhancement. The poor bioaccessibility of free St is attributable to its crystalline hydrophobicity, which causes it to precipitate during digestion and fail to integrate into dietary mixed micelles [48]. In striking contrast, the nanoencapsulated St, existing in an amorphous state, disperses readily in the aqueous intestinal phase and partitions efficiently into bile salt micelles. Furthermore, WPP-derived peptides may co-assemble with bile salts to form mixed micelles, further potentiating St solubilization [49]. This substantial improvement in bioaccessibility underscores the application potential of the St@WPP system in overcoming the inherent bioavailability bottleneck of phytosterols.

### 3.3. Self-Assembly Mechanism and Microstructural Features of St@WPP

#### 3.3.1. Non-Covalent Interaction Mapping by FTIR

**Figure 3:**
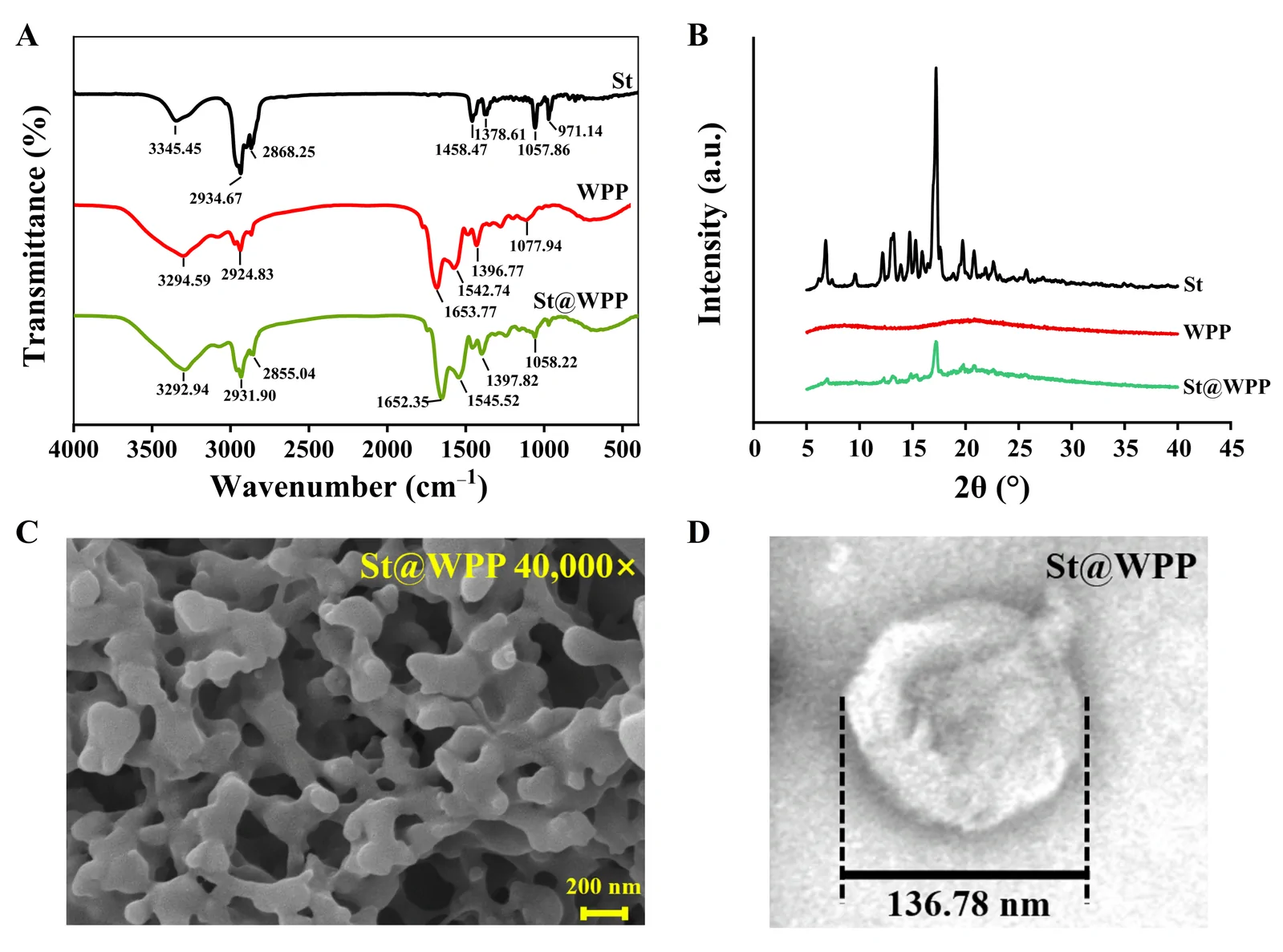
Self-assembly mechanism and microstructure of St@WPP. (**A**) FTIR spectra. (**B**) XRD patterns. (**C**) SEM and (**D**) TEM images of St@WPP.

FTIR spectroscopy was employed to elucidate the intermolecular interactions governing St encapsulation within WPP nanoassemblies. The FTIR spectra of St, WPP, and St@WPP are overlaid in Figure 3A. Free St exhibited characteristic absorption bands at 3345 cm^−1^ (O–H stretching vibration), 2850–2935 cm^−1^ (C–H_3_ and C–H_2_ stretching vibrations), 1458 cm^−1^ (C–H_2_ bending vibration), 1378 cm^−1^ (C–H_3_ bending vibration), and 1057 cm^−1^ (polycyclic ring vibration), in agreement with prior reports [50]. Upon encapsulation, the St@WPP spectrum showed markedly attenuated St-specific peaks, while the major WPP bands were largely preserved with minor shifts, indicating that St associates with WPP primarily through non-covalent interactions rather than covalent bonding. Notably, the O–H stretching peak broadened and red-shifted from 3345 to ~3293 cm^−1^ in St@WPP, which is suggestive of hydrogen bonding interactions [51]. Concurrently, diminished intensity of C–H stretching vibrations between 2800 and 3000 cm^−1^ implied the possible involvement of hydrophobic interactions in stabilizing the micellar core [52].

#### 3.3.2. XRD Analysis of St@WPP

XRD was utilized to evaluate the crystallinity and structural organization of the encapsulated St. As shown in Figure 3B, free St displayed intense crystalline diffraction peaks, with a dominant signal at 17.2° and several minor reflections between 10–25°, confirming its highly ordered crystalline nature. In contrast, WPP exhibited a broad and diffuse halo, which is characteristic of amorphous materials. Notably, the XRD pattern of St@WPP showed no sharp crystalline peaks corresponding to St, with only faint residual signals detectable in the 10–25° region. This marked reduction in crystalline diffraction suggests that St is predominantly entrapped within the micellar core, likely in a non-crystalline state, with only a negligible fraction adhering to the nanoparticle surface in crystalline form. This loss of crystalline order is mechanistically significant, as amorphous solids lack the periodic lattice structure of crystals and therefore require substantially less energy to dissolve [53]. Accordingly, the loss of crystallinity provides a thermodynamic rationale for the markedly enhanced aqueous solubility and bioaccessibility of St@WPP observed in vitro. It should be noted, however, that this interpretation warrants consideration of the potential diluting effect of WPP, which may partially attenuate the crystalline signal. Therefore, complementary techniques such as differential scanning calorimetry would be valuable for further confirming amorphization and for quantifying the extent of the crystalline-to-amorphous transition.

#### 3.3.3. Morphological Characterization of St@WPP by SEM and TEM

Peptide-based self-assemblies can adopt diverse morphologies including spheres, vesicles, tubules, or ribbons, depending on the balance of electrostatic, hydrophilic, and hydrophobic forces encoded in the amino acid sequence [54]. SEM and TEM were therefore employed to visualize the microstructure of St@WPP. SEM imaging (Figure 3C) revealed a smooth, rod-like morphology, which likely arises from the aggregation and structural collapse of nanoparticles during freeze-drying [55]. TEM (Figure 3D) further confirmed well-dispersed, spherical nanoparticles with clearly delineated boundaries, corroborating the successful encapsulation of St within intact micellar structures. The spherical morphology, combined with a uniform negative surface charge, suggests favorable attributes for epithelial uptake and intestinal transport.

### 3.4. In Vivo Hypocholesterolemic Efficacy and Multi-Target Mechanisms of St@WPP in HFD-Fed Mice

#### 3.4.1. St@WPP Alleviated HFD-Induced Obesity, Dyslipidemia, and Hepatic Injury

**Figure 4:**
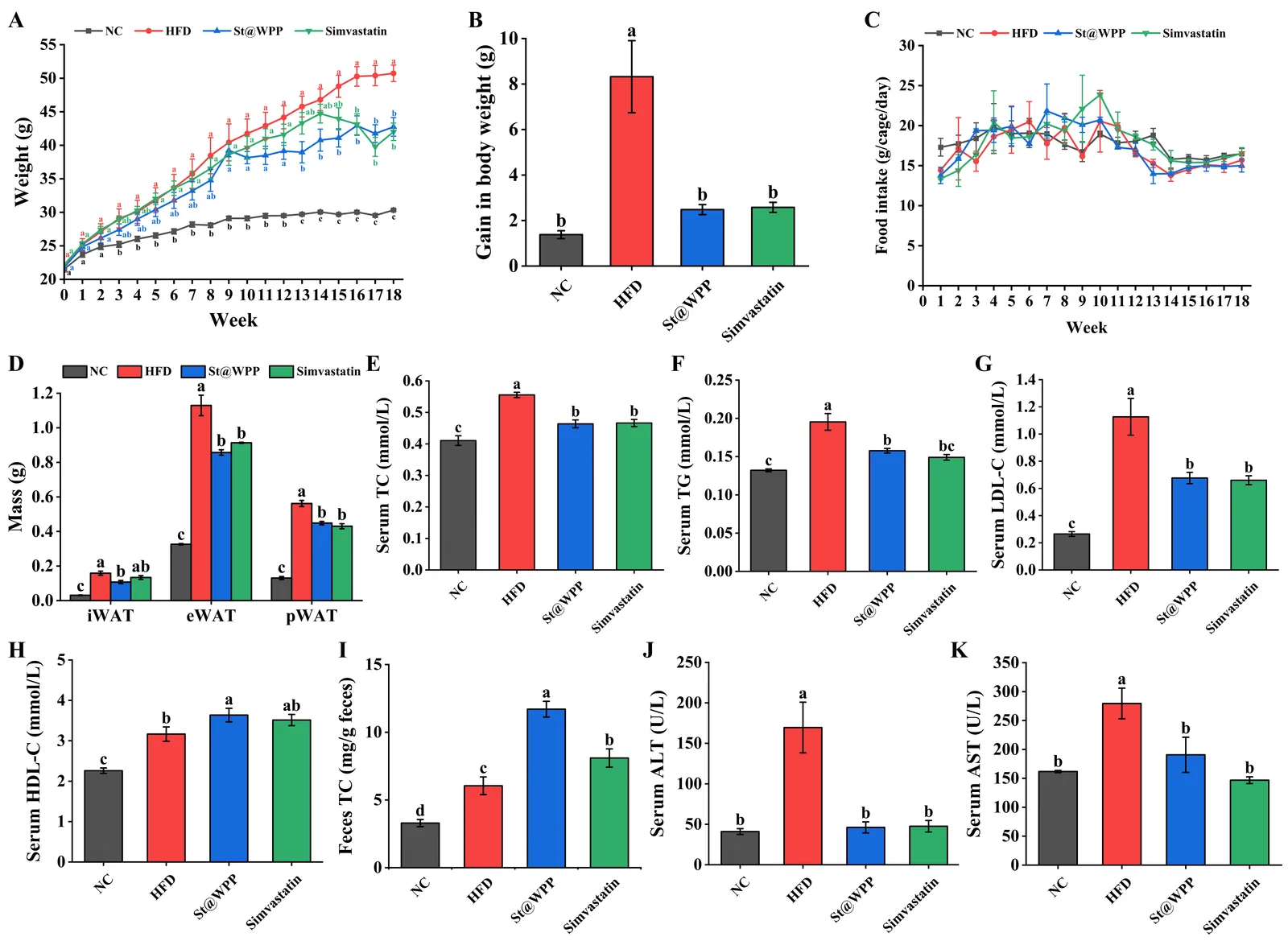
Effects of St@WPP on body weight gain, white adipose tissue mass, lipid profiles and hepatic enzyme levels in mice. (**A**) Body weight curves. (**B**) Body weight gain during the course of St@WPP administration. (**C**) Changes in food intake. (**D**) White adipose tissue mass. (**E**) Serum TC, (**F**) TG, (**G**) LDL-C, and (**H**) HDL-C. (**I**) Fecal cholesterol. (**J**) Serum ALT and (**K**) AST. Data are presented as mean ± SEM. Body weight data were analyzed using two-way repeated-measures ANOVA (group × time) with Greenhouse–Geisser correction and Bonferroni post hoc test. For food intake, *n* = 2 cages per group (5 mice per cage); for all other parameters, *n* = 6 mice per group. Different lowercase letters indicate significant differences (*p* < 0.05).

Chronic high-fat diet feeding for 17 weeks successfully induced a pronounced obese phenotype, as evidenced by significantly elevated final body weight, weight gain, and white adipose tissue (iWAT, eWAT, pWAT) mass in the HFD group compared to the NC group (Figure 4A–D, *p* < 0.05). Notably, daily gavage with St@WPP effectively counteracted these obesogenic alterations without affecting food intake (Figure 4C), suggesting that the anti-obesity effect was independent of appetite suppression.

Consistently, HFD-fed mice exhibited marked hyperlipidemia, with serum TC, TG, and LDL-C levels substantially elevated relative to the NC group (Figure 4E–H, *p* < 0.05), confirming successful establishment of the hypercholesterolemic model. St@WPP intervention significantly reduced these parameters, achieving 16.53% and 39.94% reductions in TC and LDL-C, respectively, improvements comparable to simvastatin treatment. Interestingly, serum HDL-C, which was compensatorily elevated in the HFD group, likely to facilitate reverse cholesterol transport [56], was further increased by 14.84% following St@WPP administration (Figure 4H), suggesting enhanced lipid clearance efficiency.

Fecal cholesterol content was elevated by 93.45% in the St@WPP group relative to the HFD group (Figure 4I). This marked increase in fecal cholesterol content, coupled with the reduced circulating LDL-C, may suggest that St@WPP could interfere with intestinal cholesterol absorption. This effect might be partly attributable to the competitive displacement of cholesterol from mixed micelles by St and to the CEase-inhibitory activity of the WPP shell [18,57].

#### 3.4.2. Effects of St@WPP on Serum Hepatic Enzymes

The liver centrally governs cholesterol and triglyceride metabolism to maintain systemic lipid homeostasis, and its injury typically manifests as elevated serum ALT and AST levels [27,58]. As shown in Figure 4J,K, serum AST and ALT levels were significantly higher in the HFD group than in the NC group (*p* < 0.05), confirming HFD-induced hepatic injury. Notably, St@WPP administration significantly decreased serum ALT and AST levels by 72.78% and 31.75%, respectively, relative to the HFD group (*p* < 0.05), outperforming the effect of lotus seed core powder–phytosterols treatment reported previously [28]. These results indicate that St@WPP effectively ameliorates HFD-induced liver injury, thereby supporting its regulatory role in systemic lipid metabolism.

#### 3.4.3. Alleviation of Hepatic Steatosis and Adipocyte Hypertrophy by St@WPP in HFD Mice

**Figure 5:**
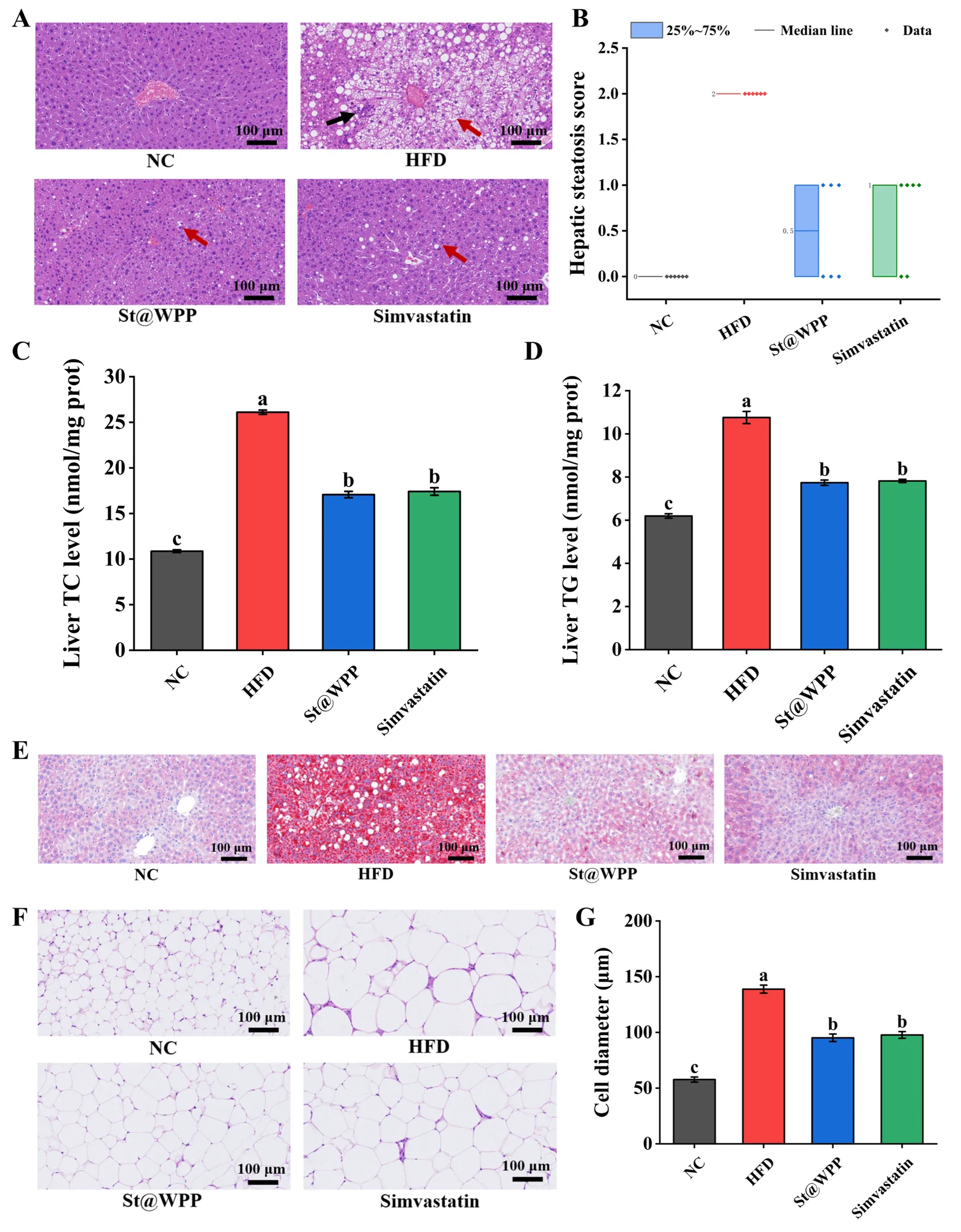
Effects of St@WPP on liver and adipose histopathology in mice. (**A**) Representative H&E stain images of the liver and (**B**) hepatic steatosis score. (**C**) Liver TC and (**D**) TG. (**E**) Representative ORO stain images of the liver. (**F**) Representative H&E stain images of the eWAT. (**G**) Adipocyte size of eWAT. The red arrow points to fat vacuoles in the cells, while the black arrow points to inflammatory cell infiltration. For ordinal data (**B**), data are presented as median (IQR). For continuous data (**C**,**D**,**G**): data are presented as mean ± SEM, *n* = 6. Different lowercase letters indicate significant differences (*p* < 0.05).

We further investigated the effects of St@WPP intervention on liver histopathology using H&E staining, as depicted in Figure 5A. Mice in the HFD group exhibited pronounced hepatic steatosis, characterized by abundant fatty vacuolation (red arrows) and inflammatory cell infiltration (black arrows)—hallmarks of non-alcoholic fatty liver disease (NAFLD). In contrast, St@WPP administration dramatically reduced hepatic fatty vacuoles and virtually eliminated inflammatory foci, corresponding to a significant reduction in the steatosis score (median (IQR): 0.5 (0, 1) vs. 2 (2, 2) for the HFD group, Figure 5B). These qualitative observations were quantitatively supported by hepatic TC and TG measurements (Figure 5C,D), both of which were significantly reduced by St@WPP relative to the HFD group (*p* < 0.05), with efficacy paralleling that of simvastatin. Oil Red O staining (Figure 5E) visually confirmed the apparent attenuation of intracellular lipid accumulation in St@WPP-treated hepatocytes.

Under normal physiological conditions, hepatic cholesterol homeostasis is maintained through reverse cholesterol transport mediated by high-density lipoprotein (HDL), which transports cholesterol to the liver for subsequent excretion into bile and elimination via feces [59]. HFD-induced hypercholesterolemia, however, leads to excessive hepatic lipid deposition, disrupts hepatic cholesterol homeostasis, and impairs bile acid synthesis and cholesterol excretion pathways, ultimately resulting in elevated blood lipid levels [60,61]. The observed reduction in hepatic lipids by St@WPP may indicate a restoration of reverse cholesterol transport capacity, which underpins the improved serum lipid profile. Furthermore, hepatic steatosis stimulates the release of pro-inflammatory factors such as TNF-α, IL-6, and IL-1β, contributing to liver inflammation and cellular injury, aligning with our observations [62]. These results suggest that St@WPP alleviates hypercholesterolemia by regulating intrahepatic lipid metabolism and transport, thereby reducing lipid accumulation and inflammatory responses.

Beyond the liver, adipose tissue serves as a primary energy reservoir and a key endocrine organ that modulates systemic lipid metabolism via secretion of various adipokines [63]. H&E staining of epididymal white adipose tissue (eWAT, Figure 5F,G) revealed that HFD feeding induced adipocyte hypertrophy, with cells exhibiting irregular architecture and significantly enlarged diameters compared to the NC group (*p* < 0.05). St@WPP intervention effectively reversed this hypertrophy, reducing the average adipocyte diameter by 31.46% (*p* < 0.05) and restoring a more homogeneous tissue organization with reduced intercellular spaces. Collectively, these histopathological improvements affirm that St@WPP not only lowers circulating lipids but also alleviates ectopic lipid accumulation in both hepatic and adipose depots.

#### 3.4.4. Attenuation of Systemic Inflammation by St@WPP in HFD Mice

**Figure 6:**
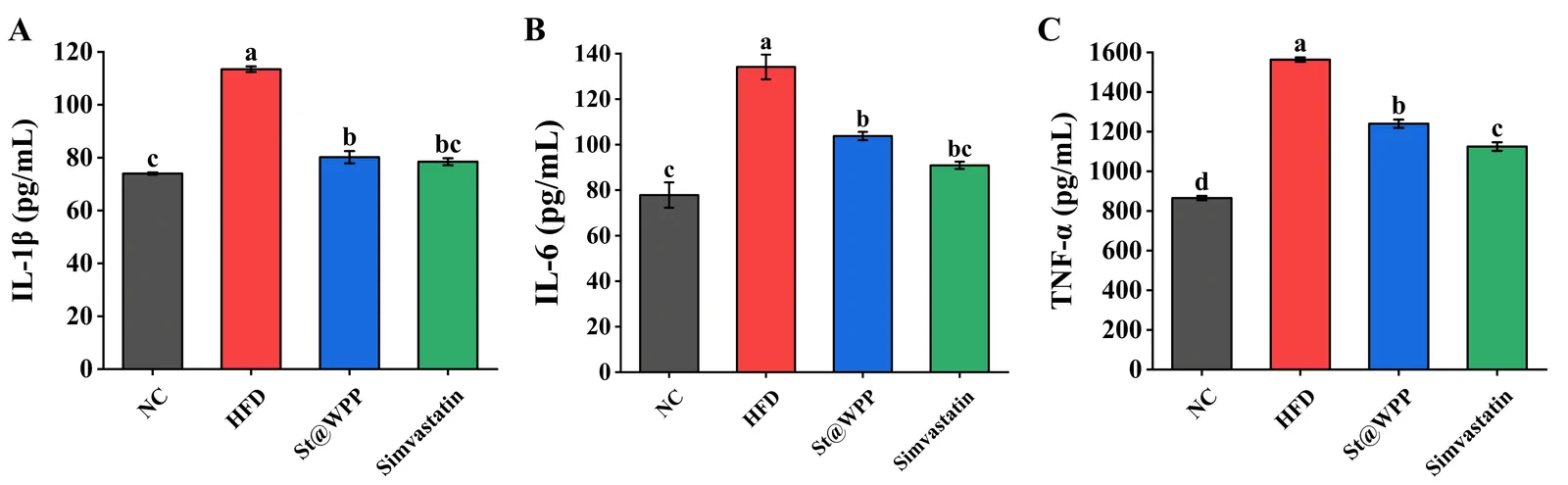
Effects of St@WPP on serum inflammatory cytokines in mice. (**A**) Serum IL-1β, (**B**) IL-6, and (**C**) TNF-α. Data are presented as mean ± SEM, *n* = 6. Different lowercase letters indicate significant differences (*p* < 0.05).

Given that lipid overload triggers pro-inflammatory cascades, we measured serum cytokines (Figure 6A–C). HFD feeding significantly elevated serum IL-1β, IL-6, and TNF-α levels, whereas St@WPP administration markedly reduced these mediators to levels approaching those in the NC group (*p* < 0.05). This anti-inflammatory effect is mechanistically intertwined with reduced hepatic steatosis and adipocyte size, as hypertrophic adipocytes are known to be major sources of inflammatory adipokines. By limiting lipid accumulation, St@WPP indirectly attenuates the obesity-driven low-grade systemic inflammation.

#### 3.4.5. St@WPP Remodels Gut Microbiota Composition and Short-Chain Fatty Acids (SCFAs) Metabolism in HFD Mice

**Figure 7:**
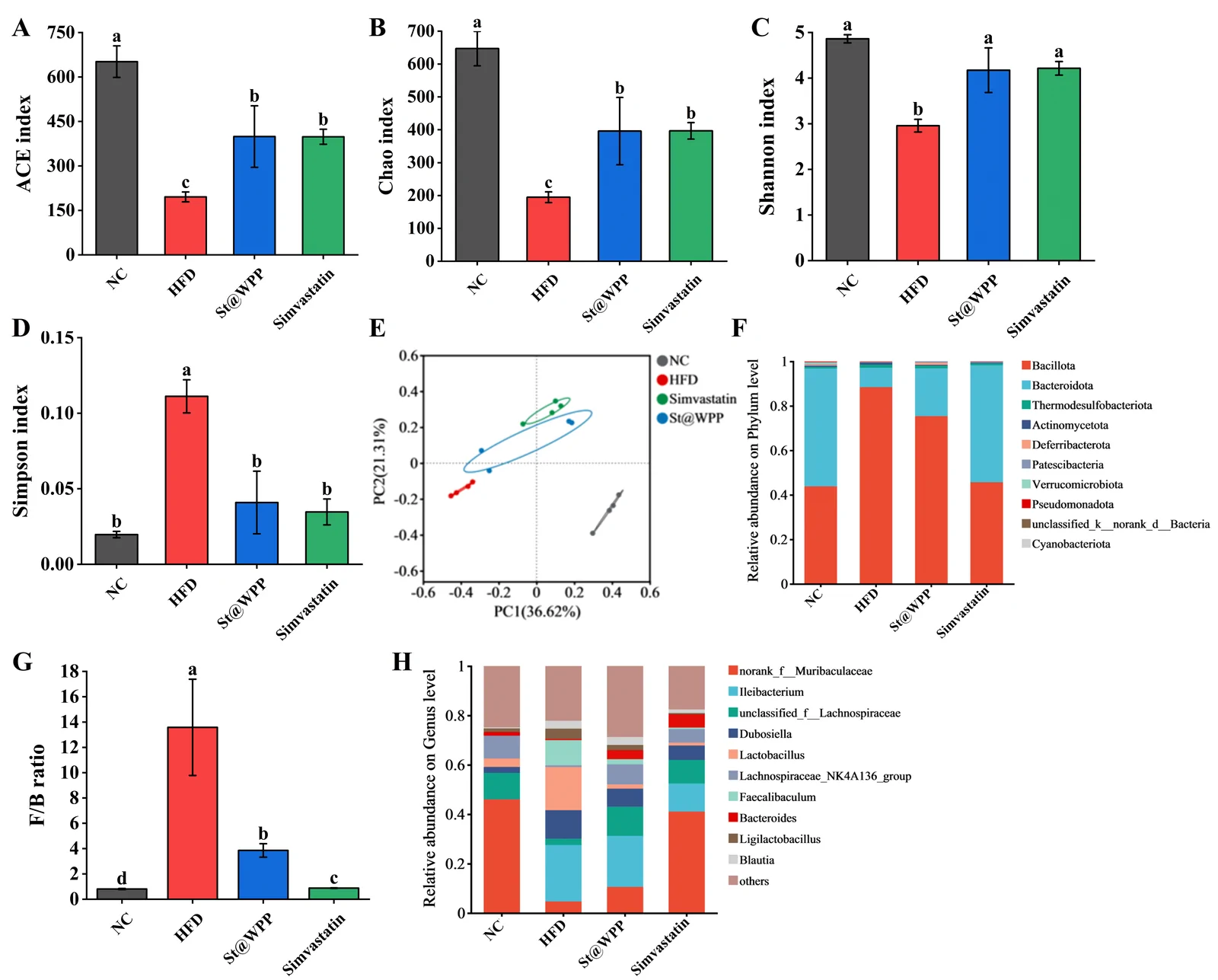
Effects of St@WPP on gut microbiota diversity, richness, and structure in mice. (**A**) ACE index, (**B**) Chao index, (**C**) Shannon index, and (**D**) Simpson index (α-diversity). (**E**) PCoA of β-diversity. (**F**) Bacterial composition at the phylum level. (**G**) F/B ratio. (**H**) Bacterial composition at the genus level. Data are presented as mean ± SEM, *n* = 6. Different lowercase letters indicate significant differences (*p* < 0.05).

The pronounced hypocholesterolemic efficacy of St@WPP prompted us to investigate its impact on the gut ecosystem, given that hyperlipidemia profoundly disrupts gut microbial homeostasis and intestinal barrier function [64]. As shown in Figure 7A–D, chronic HFD feeding significantly decreased alpha-diversity indices (ACE, Chao, and Shannon) while elevating the Simpson index, indicative of lower microbial richness and evenness. Notably, St@WPP intervention significantly counteracted these negative shifts, shifting diversity toward levels approaching those of the NC group. Principal coordinates analysis (PCoA) was used to analyze the differences and similarities in gut microbiota composition among the various treatment groups. As shown in Figure 7E, a significant separation was observed between the NC group and the high-fat diet groups (HFD, St@WPP, and Simvastatin group). PCoA revealed a distinct compositional divergence between the NC and HFD clusters, whereas the St@WPP and simvastatin groups converged towards the NC configuration (R^2^ = 0.6131, *p* = 0.001). This restructuring suggests that St@WPP effectively restored the disrupted microbial community towards a healthier equilibrium. Differences in gut microbiota composition among various treatment groups at the phylum and genus levels are illustrated in Figure 7F–H. The St@WPP intervention reversed the significant increase in the relative abundance of Firmicutes and the significant decrease in the relative abundance of Bacteroidetes induced by a high-fat diet, resulting in a 71.60% reduction in the F/B ratio (*p* < 0.05, Figure 7G). An abnormally elevated F/B ratio is commonly associated with gut microbiota dysbiosis related to obesity, fat deposition, and lipid metabolism disorders [65]. Additionally, compared to the HFD group, the St@WPP intervention significantly reduced the relative abundances of *Ileibacterium*, *Lactobacillus*, *Faecalibaculum*, and *Blautia*, while increasing the relative abundances of *norank_f__Muribaculaceae*, *Lachnospiraceae_NK4A136_group*, *Bacteroides*, and *unclassified_f__Lachnospiraceae*, consistent with previous studies [66,67]. These results indicate that a high-fat diet significantly reduced the species richness and diversity of the gut microbiota, while St@WPP intervention effectively ameliorated this dysbiotic state, promoting a shift toward a normal microbiota structure. We speculate that this may be attributed to nanoparticle formation, which enhances the bioaccessibility of St, allowing more St to reach the intestine and be utilized by the gut microbiota, thereby improving the gut microbial structure [28].

**Figure 8:**
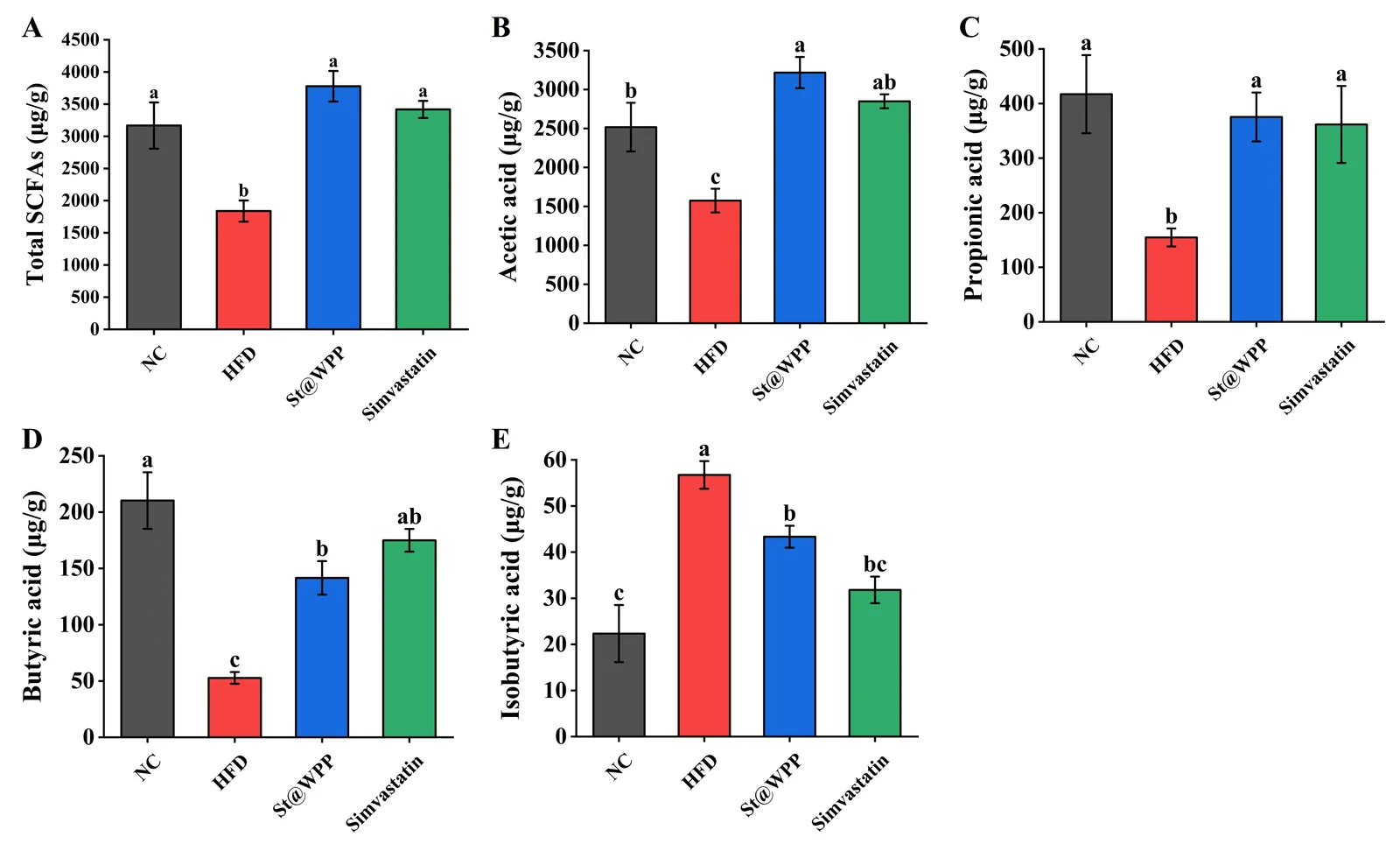
Effects of St@WPP on fecal SCFAs content in mice. (**A**) Total SCFAs, (**B**) acetic acid, (**C**) propionic acid, (**D**) butyric acid, and (**E**) isobutyric acid. Data are presented as mean ± SEM, *n* = 6. Different lowercase letters indicate significant differences (*p* < 0.05).

SCFAs, including acetate, propionate, and butyrate, are metabolites produced by gut microbiota. The levels of SCFAs are associated with improvements in obesity, lipid metabolism, and inflammatory responses [68]. As shown in Figure 8A–E, the St@WPP interventions significantly reversed the decrease in total SCFAs, acetic acid, propionic acid, and butyric acid levels caused by the high-fat diet (*p* < 0.05). Notably, the level of isobutyric acid, which is positively correlated with obesity and metabolic disorders, was significantly lower in the St@WPP group compared to the HFD group [69]. These findings may be attributed to structural changes in the gut microbiota that increased the abundance of bacteria and metabolic pathways responsible for producing SCFAs, leading to increased SCFA production in the gut.

### 3.5. Limitations and Future Directions

Although the present study focused on the preparation and characterization of St@WPP and confirmed its in vivo cholesterol-lowering efficacy, the underlying mechanisms, particularly the regulatory pathways and the relative contributions of each component, require further elucidation. Future studies should include assessment of the CEase inhibitory activity of both the peptides identified by molecular docking and the assembled St@WPP formulation, direct evaluation of intestinal cholesterol absorption, and analysis of fecal sterol excretion kinetics. Additionally, control groups treated with free St, free WPP, and their physical mixture would help to determine whether the encapsulated formulation exerts synergistic effects beyond the mere additive actions of the individual components. Furthermore, it should be noted that the present study did not establish a causal relationship between gut microbiota changes and the observed hypocholesterolemic effects. Thus, to further elucidate the impact of hypercholesterolemia on gut microbiota and microbiota-mediated cholesterol-lowering mechanisms, future investigations incorporating Kyoto Encyclopedia of Genes and Genomes (KEGG) pathway prediction, correlation analysis between gut microbiota and host lipid parameters, and fecal microbiota transplantation experiments are warranted.

## 4. Conclusions

In conclusion, this study successfully reports a stigmasterol-loaded nanoplatform (St@WPP) assembled from whey protein-derived peptides with CEase-inhibitory properties. The self-assembled nanoparticles, driven by hydrophobic and hydrogen-bonding interactions, convert crystalline stigmasterol into an amorphous nanoformulation, markedly improving its aqueous dispersibility and bioaccessibility. Notably, St@WPP demonstrated improved cholesterol-lowering efficacy, as evidenced by reduced serum LDL-C, alleviated hepatic steatosis, suppressed systemic inflammation, and restored gut microbial homeostasis in HFD-fed mice. The observed hypocholesterolemic effects may involve modulation of cholesterol absorption and excretion, which contributes to the reduced lipid accumulation in the serum, liver, and adipocytes. This finding provides a promising strategy for improving the bioaccessibility of stigmasterol and supports its application in functional foods aimed at regulating lipid metabolism.

## Data Availability

The original contributions presented in this study are included in the article/Supplementary Materials. Further inquiries can be directed to the corresponding author.

## Supplementary Materials

The following supporting information can be downloaded at https://www.mdpi.com/article/10.3390/nu18172934/s1, Table S1: Formula for stock solution of simulated gastric fluid (SGF) and simulated intestinal fluid (SIF) (adult); Table S2: Ingredients of normal diet and high-fat diet; Table S3: Sequence of potential biologically active peptides and their interaction with cholesterol esterase (PDB code: 1CLE-chain: A) binding sites; Table S4: Polydispersity index (PDI) values of St@WPP at varying St:WPP mass ratios.

## Abbreviations

HMGCR	3-Hydroxy-3-methylglutaryl-coenzyme A reductase
ACAT	Acyl-CoA:cholesterol acyltransferase
St	Stigmasterol
Ps	Phytosterols
WPP	Whey protein peptides
SDS	Sodium dodecyl sulfate
OPA	Ortho-phtaldialdehyde
DTT	Dithiothreitol
PNPB	4-Nitrophenyl butyrate
CEase	Cholesterol esterase
PL	Pancreatic lipase
TG	Triglyceride
TC	Total cholesterol
IL-1β	Interleukin-1β
IL-6	Interleukin-6
TNF-α	Tumour necrosis factor-α
AST	Aspartate aminotransferase
ALT	Alanine aminotransferase
LDL-C	Low-density lipoprotein cholesterol
HDL-C	High-density lipoprotein cholesterol
St@WPP	St-loaded WPP nanoparticles
DLS	Dynamic light scattering
H&E	Hematoxylin or eosin
PCoA	Principal coordinate analysis
DH	Degree of hydrolysis
CMC	Critical micelle concentration
LC	Loading capacity
EE	Encapsulation efficiency
FTIR	Fourier-transform infrared
XRD	X-ray diffraction
SEM	Scanning electron microscopy
TEM	Transmission electron microscopy
HFD	High-fat diet
eWAT	Epididymal white adipose tissue
pWAT	Perirenal white adipose tissue
iWAT	Inguinal white adipose tissue
NAFLD	Non-alcoholic fatty liver disease
HDL	High-density lipoprotein
SCFAs	Short-chain fatty acids
