# Supplementary material for: Whey Protein Peptides Self-Assembled Nanoparticles with Intrinsic Cholesterol Esterase Inhibition Enhance Stigmasterol Bioaccessibility and Hypocholesterolemic Effects
Source: Nutrients. 2026 Sep 7;18(17):2934. doi: 10.3390/nu18172934 (PMC13567727; doi:10.3390/nu18172934)
Supplement: Supplementary file 1 [file nutrients-18-02934-s001.zip › nutrients-4494328-supplementary.pdf]

***In vitro* gastrointestinal digestion**

The samples were passed through a two-stage gastrointestinal model, including the stomach phase and small intestinal phase *in vitro*. Simulated gastric fluid (SGF) and simulated intestinal fluid (SIF) were prepared as detailed in Table S1.

Initial system: 50 mL of free St and St@WPP sample solution containing equal amounts of St were stirred at 37°C for 30 min.

Simulated stomach phase: 50 mL of the initial sample was transferred into a 100 mL beaker and mixed with 50 mL of SGF containing 16 mg/mL pepsin and 25 μL $CaCl_2$. The mixture was then adjusted to pH 3.0 and continuously stirred for 2 h at 37°C.

Simulated gastrointestinal phase: 50 mL of the stomach phase sample was transferred into a 100 mL beaker, where the pH was adjusted to 7.0. Subsequently, 0.1 mL of $CaCl_2$ and 50 mL of SIF solution (containing bile salts, 2500 mg pancreatin and 1520 mg pancreatic lipase) were added. The samples were titrated with 1 M NaOH to maintain a constant pH 7.0 at 37°C for 2 h.

After the small intestine digestion, the digestive solution was inactivated by heating at 100°C for 20 min.

Table S1 Formula for stock solution of simulated gastric fluid (SGF) and simulated intestinal fluid (SIF) (adult).

	**SGF（pH = 3）（mL）**	**SIF（pH = 7）（mL）**
0.5 mol/L KCl	6.9	6.8
0.5 mol/L KH_2PO_4	0.9	0.8

1 mol/L $NaHCO_3$	12.5	42.5
2 mol/L NaCl	11.8	9.6
0.15 mol/L $MgCl_2(H_2O)_6$	0.4	1.1
0.5 mol/L $(NH_4)_2CO_3$	0.5	—
6 mol/L HCl	1.3	0.7
1 mol/L NaOH	—	—
0.3 mol/L $CaCl_2(H_2O)_2$	—	—
Final adjustment	adjust to 500 mL with deionized water	

Table S2 Ingredients of normal diet and high-fat diet.

Ingredients (g/kg diet)	Normal diet	High-fat diet
Casein, Lactic, 30 Mesh	189.56	258.45
Cystine, L	2.84	3.88
Starch, Corn	479.79	-
Lodex 10	118.48	161.53
Sucrose, Fine Granulated	69.00	94.08
Solka Floc, FCC200	47.39	64.61
Lard	18.96	316.60
Soybean Oil, USP	23.70	32.31
Mineral Mix S10026B	47.39	64.61
Choline Bitartrate	1.90	2.58
Vitamin Mix V10001C	0.95	1.29
Dye	0.05	0.06

Identification of whey protein-derived cholesterol esterase inhibitory peptides

The whey protein peptides were dissolved in a 0.1% formic acid solution and analyzed using an Orbitrap Q Exactive™ instrument connected to a Dionex Ultimate 3000 HPLC system (Thermo Fisher Scientific, MA, USA). The LCMS grade acetonitrile containing 0.1% formic acid (solvent B) and deionized water consisting of 0.1% formic acid (solvent A) was used as mobile phase. Peptide separation was conducted using an Accucore™ C18 column (2.1 mm×100 mm×2.6 μm particles) at a flow rate of 300 μL/min and a column temperature of 40°C. The mass spectrometer was operated with the following parameters: collision energy of 28%, mass range spanning from 200 to 2000 m/z, and ion source voltage set at 2 kV. Raw data were processed using Proteome Discoverer (v2.4). Database searches were performed against the UniProt database with the following parameters: precursor ion mass tolerance of 10 ppm, fragment ion mass tolerance of 0.1 Da, carbamidomethylation of cysteine as a fixed modification, and oxidation of methionine as a variable modification. Peptide identifications were filtered at a 1% false discovery rate (FDR) at the PSM, peptide, and protein levels, and only high-confidence peptides (FDR < 1%) were retained for further analysis.

Peptide toxicity predictions were conducted using the ToxinPrep platform (https://webs.iiitd.edu.in/raghava/toxinpred/index.html). Allergenicity assessments were carried out with AllerCatPro 2.0 (https://allercatpro.bii.a-star.edu.sg). Human intestinal absorption was evaluated using admetSAR (https://lmmd.ecust.edu.cn/admetsar1/home). Peptide novelty analysis was performed

through BIOPEP-UWM (https://biochemia.uwm.edu.pl/biopep-uwm).

Table S3 Sequence of potential biologically active peptides and their interaction with cholesterol esterase (PDB code: 1CLE-chain: A) binding sites.

**Peptide**	**Parent Whey Protein**	**Sequence Position**	**Toxicity**	**Allergenicity**	**Human Intestinal Absorption**	**- CDOCKER ENERGY (kcal/mol）**	**Bound residue in CEase (PDB ID: 1CLE-chain: A)**
DTDYK	β-lactoglobulin	112–116	Nontoxic	No	0.5536	97.7155	Gly123, Gly124, Glu208, Ser209, Phe296, Leu302, Val344, Ala450
KDLK	α-Lactalbumin	32–35	Nontoxic	No	0.7548	91.3308	Gly123, Gly124, Glu208, Phe296, Val344, His449
TMKGL	β-lactoglobulin	22–26	Nontoxic	No	0.5475	88.2546	Pro65, Gly122, Gly124, Phe125, Ile127, Glu208, Ser209, Ala210, Met213, Leu302. Leu304, Ala414, His449, Ala450
KGYGGV	α-Lactalbumin	35–40	Nontoxic	No	0.6847	82.0885	Gly122, Gly123, Gly124, Glu208, Ser209, Ala210, Met213, Leu302. Leu304, Phe345, Phe415, Met445, Phe448, His449

KIDAL	β-lactoglobulin	99–103	Nontoxic	No	0.5427	80.9911	Gly123, Gly124, Phe125, Ile127, Ser209, Ala210, Met213, Phe296, Leu302, Ala414, Phe415, His449

Table S4 Polydispersity index (PDI) values of St@WPP at varying St:WPP mass ratios

**St:WPP mass ratio**	**PDI**
1.6:20	0.15±0.01
3.2:20	0.19±0.01
6.4:20	0.12±0.02
8.0:20	0.28±0.04

Data are presented as mean ± SD (n=3).
